# Blood pressure response to clonidine in children with short stature is correlated with postural characteristics: a retrospective cross-sectional study

**DOI:** 10.1186/s12887-023-04506-z

**Published:** 2024-01-13

**Authors:** Wentao Yang, Shanshan Wang, Wei Gu, Francis Manyori Bigambo, Yubing Wang, Xu Wang

**Affiliations:** 1 Department of Endocrinology, Children’s Hospital of Nanjing Medical University, 72 Guangzhou Rd, Nanjing, 210008 China; 2 Department of Emergency, Pediatric Intensive Care unit, Children’s Hospital of Nanjing Medical University, Nanjing, 210008 China; 3 Medical Clinical Research Center, Children’s Hospital of Nanjing Medical University, Nanjing, 210008 China

**Keywords:** Clonidine, Hypotension, Passive leg raising, Posture, Short stature

## Abstract

**Background:**

Clonidine stimulation test has been widely used in the diagnosis of growth hormone deficiency in children with short stature with a high level of reliability. However, it may cause hypotension, which usually appears as headache, dizziness, bradycardia, and even syncope. It is well known that elevating the beds to make patients’ feet above their cardiac level might relieve this discomfort. However, the real efficiency of this method remains to be proved while the best angle for the elevated bed is still unclear.

**Methods:**

A total of 1200 children with short stature were enrolled in this retrospective cross-sectional study. Age, gender, weight, and basic systolic and diastolic blood pressure were collected. Blood pressure at 1, 2, 3, and 4 h after stimulation tests were recorded. The participants were divided into 3 groups based on the angles of the elevated foot of their beds named 0°, 20°, and 40° groups.

**Results:**

At one hour after the commencement of the tests, participants lying on the elevated beds showed a higher mean increase on the change of pulse pressure. The difference in the angles of the elevated beds did not show statistical significance compared with those who did not elevate their beds (0.13 vs. 2.83, *P* = 0.001; 0.13 vs. 2.18, *P* = 0.005; 2.83 vs. 2.18, *P* = 0.369). When it came to 4 h after the tests began, participants whose beds were elevated at an angle around 20° had a significantly higher mean increase in the change of pulse pressure values compared with those whose beds were elevated at an angle around 40° (1.46 vs. -0.05, *P* = 0.042).

**Conclusion:**

Elevating the foot of the beds of the patients who are undergoing clonidine stimulation tests at an angle of 20°might be a good choice to alleviate the hypotension caused by the tests.

## Introduction

With development of the economy and arousal of the attention paid to body stature, there is an increasing sought for early medical treatment of children with short stature. Normally, short stature is defined as a height more than two standard deviations (SD) below the mean height of the reference population matched for age, sex, and pubertal stage [1]. This meant that about 2.3% of the population would be diagnosed as short stature. Growth hormone deficiency is a common reason for the short stature of these children with an estimated prevalence of 1 patient per about 4000-10,000 children [2]. To confirm whether growth hormone is lacking in a timely and precise is of great importance, which highly depends on stimulation tests [3].

Clonidine stimulation test (CST) has been seen as a common way to evaluate the ability of children to secrete growth hormone [4]. However, the side effect of this test may be a reduction of blood pressure of the tested children to a dangerous range, which has always been a disturbing problem, leading to headaches, dizziness, bradycardia, and even syncope [5].

Body posture has been confirmed to have effects on blood pressure [6]. Passive leg raising (PLR) used to be recommended as the initial treatment of shock and hypotension [7]. This posture refers to raising the legs above cardiac level, with the patient in a supine position, and has been thought to help increase the volume of the returned blood, which may therefore raise the blood pressure as well as the pulse pressure [8]. Due to the possible effect of PLR, some treatment groups in our hospital tried PLR in clinical practice and found that raising half of the bed when a child is taking CST and keeping a child lying on the bed until 4 h after the test began was just the same as PLR. This seemed to be an effective way to deal with hypotension caused by CST. Typically, PLR was thought to be a transient effect, which may only last for a few minutes. However, a meta-analysis indicated that PLR seemed to have long-term effects of increasing cardiac output, which may last for at least 10 min [9]. Also, a study made in Iran indicated that PLR performed 2 min before anesthesia induction and continued for 20 min after tracheal intubation can help decrease the incidence of anesthesia-induced hypotension 20 min after intubation [10]. Although these studies are not relevant to the current study population, they all indicated the potential long-term effects of PLR.

Whether PLR can help reduce the problems of hypotension caused by clonidine has not yet been proven and the best angle to raise the body still needs to be confirmed. Therefore, in this retrospective cross-sectional study, we aimed to find whether postural characteristics, such as PLR, can be used to help relieve the side effects of CST and to find the best angle raised in these tests, which can better relieve the hypotension of the tested children.

## Methods

From September 2020 to March 2022, children from several treatment groups who were hospitalized at the Children’s Hospital of Nanjing Medical University to participate in the clonidine stimulation tests were collected in this retrospective cross-sectional study. Only children with a height of two SD below the mean height of the reference population matched for age, sex, and pubertal stage were included in the study. Subjects with SBP higher than 140 mmHg or lower than 65 mmHg and with DBP higher than 100 mmHg or lower than 35 mmHg were excluded. All participants provided written informed consent to have their clinical information analyzed in a clinical study. All in all, we included 1200 children in this study.

Basic systolic and diastolic blood pressure (SBP, DBP) in the supine position were measured by trained nurses. Changes in blood pressure at 1,2,3 and 4 h of the CST were recorded. Pulse pressure (PP) was calculated by using the formula: PP = SBP- DBP. ΔSBP, ΔDBP, and ΔPP were calculated by using the values of blood pressure at each time to minus the values of their baseline blood pressure. The dosage of the clonidine used in the stimulation test was 4 g/kg (no more than 150 g). Blood was drawn every 30 min for four times. The total time of the test was 90 min but the children would be kept lying on beds for at least 4 h for safety.

During the whole test, children were kept lying on their elevated beds and the angles of these beds were set by nurses. Different treatment groups tended to use different angles. According to the angle of the beds they lay on, the tested children were divided into three groups namely the 0° group (N = 346), 20° group (N = 354), and 40° group (N = 500).

Four covariates including age, gender, weight, and the dosage of clonidine were considered in this analysis. One-way ANOVA was used to compare differences in age, weight, the dosage of clonidine, and blood pressure changes one hour after CST began between the three groups. LSD was used as a post hoc test for results with statistically significant differences after One-way ANOVA. The chi-square test was used to compare the difference in gender between the three groups. The t-test was used to compare the differences in blood pressure at two, three, and four hours after CST began between the 20° and 40° groups. All analyses were performed with SPSS statistical software (Version 26.0. Armonk, NY: IBM Corp).

## Results

This study included 1200 children from Nanjing, China who were divided into three groups namely 0°, 20°, and 40° groups. The distributions of blood pressure of these three groups at different periods are presented by mean ± SD, percentile, and range in Table 1. Generally, drops in blood pressure levels were observed during the first 2 h after CST began, and then from 3 to 4 h after the commencement of CST, blood pressure levels gradually recovered.

**Table 1:** Distribution of blood pressure in different positions at different time periods

	Mean ± SD	25th	50th	75th	95th	Range
0° (*N* = 346)						
*0 h*						
Systolic blood pressure (mmHg)	104.14 ± 10.15	97.00	104.00	110.00	121.00	71–133
Diastolic blood pressure (mmHg)	63.58 ± 9.45	58.00	64.00	70.00	78.65	41–96
Pulse pressure (mmHg)	40.57 ± 7.70	36.00	40.00	44.00	55.00	22–74
*1 h*						
Systolic blood pressure (mmHg)	94.38 ± 8.93	88.00	94.00	99.25	110.00	70–126
Diastolic blood pressure (mmHg)	53.68 ± 7.32	50.00	52.00	57.00	68.00	36–79
Pulse pressure (mmHg)	40.70 ± 6.97	36.00	40.00	45.00	52.00	20–60
20° (*N* = 354)						
0 h						
Systolic blood pressure (mmHg)	104.18 ± 9.63	97.75	104.00	111.00	121.00	83–132
Diastolic blood pressure (mmHg)	62.66 ± 8.74	56.00	62.00	68.00	78.00	37–88
Pulse pressure (mmHg)	41.52 ± 7.52	37.00	41.00	46.00	54.00	15–68
*1 h*						
Systolic blood pressure (mmHg)	95.72 ± 7.93	90.00	95.00	100.00	110.00	76–122
Diastolic blood pressure (mmHg)	51.37 ± 8.24	46.00	51.00	56.25	65.25	33–77
Pulse pressure (mmHg)	44.35 ± 7.61	39.00	44.00	49.00	58.00	21–65
*2 h*						
Systolic blood pressure (mmHg)	92.19 ± 9.03	86.00	92.00	97.00	108.00	69–127
Diastolic blood pressure (mmHg)	49.58 ± 8.30	43.00	49.00	55.00	65.25	30–81
Pulse pressure (mmHg)	42.60 ± 8.02	37.00	43.00	48.00	55.25	17–71
*3 h*						
Systolic blood pressure (mmHg)	93.62 ± 10.00	87.00	93.00	99.00	112.00	62–132
Diastolic blood pressure (mmHg)	50.10 ± 8.52	44.00	50.00	55.00	67.00	30–78
Pulse pressure (mmHg)	43.52 ± 8.20	39.00	44.00	48.00	57.00	17–72
*4 h*						
Systolic blood pressure (mmHg)	96.55 ± 8.98	91.00	96.00	102.00	112.00	70–129
Diastolic blood pressure (mmHg)	53.58 ± 8.13	49.00	53.00	59.00	66.00	32–85
Pulse pressure (mmHg)	42.98 ± 7.97	38.00	43.00	48.00	56.00	20–70
40° (*N* = 500)						
*0 h*						
Systolic blood pressure (mmHg)	104.99 ± 10.43	98.00	104.00	112.00	123.00	68–138
Diastolic blood pressure (mmHg)	63.51 ± 8.38	58.00	63.00	69.00	78.95	42–87
Pulse pressure (mmHg)	41.48 ± 8.62	36.00	41.00	46.00	56.95	18–71
*1 h*						
Systolic blood pressure (mmHg)	95.95 ± 9.39	89.00	95.00	101.00	114.00	76–131
Diastolic blood pressure (mmHg)	52.29 ± 9.58	46.00	51.00	58.00	70.00	30–97
Pulse pressure (mmHg)	43.66 ± 8.26	38.00	43.00	48.00	58.00	14–77
*2 h*						
Systolic blood pressure (mmHg)	93.65 ± 8.46	88.00	93.00	99.00	109.00	73–124
Diastolic blood pressure (mmHg)	50.14 ± 8.02	45.00	50.00	55.00	64.95	27–89
Pulse pressure (mmHg)	43.52 ± 8.63	38.00	43.00	49.00	59.00	8–74
*3 h*						
Systolic blood pressure (mmHg)	94.90 ± 9.05	89.00	94.00	100.00	110.00	75–153
Diastolic blood pressure (mmHg)	50.81 ± 7.93	45.00	50.00	56.00	64.00	32–93
Pulse pressure (mmHg)	44.09 ± 8.17	39.00	43.00	49.00	59.00	15–69
*4 h*						
Systolic blood pressure (mmHg)	97.24 ± 8.04	92.00	97.00	101.00	112.00	72–129
Diastolic blood pressure (mmHg)	55.80 ± 7.02	50.25	56.00	60.00	67.00	35–80
Pulse pressure (mmHg)	41.43 ± 7.76	37.00	41.00	46.00	54.95	19–73

Age, gender, weight, doses of clonidine, basic SBP, basic DBP, and basic PP at admission were collected to compare the baseline of these three groups. During admission, no statistical differences were found in the distributions of age (P = 0.737), gender (P = 0.658), weight (P = 0.788), doses of clonidine used (P = 0.948), basic SBP (P = 0.293), basic DBP (P = 0.380) and basic PP (P = 0.195) between these three groups (Table 2).

**Table 2:** Comparison of age, gender and basal blood pressure in children with different positions at admission

	0°	20°	40°	*F*/χ^2^	*P*
Age (y)	7.66 ± 2.507	7.60 ± 2.649	7.52 ± 2.626	0.305	0.737
Gender				0.838	0.658
Male	182	189	278		
Female	164	165	222		
Weight (kg)	22.39 ± 7.56	22.65 ± 7.97	22.28 ± 7.66	0.238	0.788
Dosage (µg)	88.69 ± 27.38	88.91 ± 28.08	88.29 ± 28.54	0.053	0.948
Diastolic blood pressure (mmHg)	63.58 ± 9.453	62.66 ± 8.743	63.28 ± 8.810	1.228	0.293
Systolic blood pressure (mmHg)	104.14 ± 10.150	104.18 ± 9.632	104.99 ± 10.433	0.967	0.380
Pulse pressure (mmHg)	40.57 ± 7.704	41.52 ± 7.523	41.48 ± 8.619	1.637	0.195

One hour after the CST began, a decrease in SBP and DBP was observed in all three groups compared with their basic ones while the PP witnessed slight increases (Table 3). The results showed that both ΔSBP (P = 0.165) and ΔDBP (P = 0.322) revealed no statistical differences between these three groups. However, ΔPP values (P = 0.001) showed a statistically significant difference. By post hoc comparison, we found that the difference in ΔPP between the 0° and 20° groups (P = 0.001) as well as the difference between the 0° group and 40° group (P = 0.005) were statistically significant. The difference between 20° group and 40° group was statistically insignificant (P = 0.369). This indicated that PLR can affect the patient’s blood pressure during the tests.

**Table 3:** Comparison of blood pressure changes at 1 h after clonidine stimulation test in children with different positions

	0°	20°	40°	*F*	*P*
Δ diastolic blood pressure (mmHg)	-9.90 ± 10.54	-11.30 ± 10.93	-11.22 ± 11.83	1.805	0.165
Δ systolic blood pressure (mmHg)	-9.77 ± 11.498	-8.47 ± 10.321	-9.03 ± 12.13	1.135	0.322
Δ pulse pressure (mmHg) ^*, †, ‡^	0.13 ± 9.67	2.83 ± 9.58	2.18 ± 11.30	6.594	0.001

*. The difference in pulse pressure changes between 0° group and 20° group was statistically significant (P = 0.001)

†. The difference in pulse pressure changes between 0° group and 40° group was statistically significant (P = 0.005).

‡. The difference in pulse pressure changes between 20° group and 40° group was statistically insignificant (P = 0.369).

Table 4 shows that, two hours after CST began, no statistical differences were found in ΔSBP (P = 0.415), ΔDBP (P = 0.692), and ΔPP (P = 0.209) between the 20° and 40° groups. Then, three hours after CST began, there were also no statistical differences found in ΔSBP (P = 0.579), ΔDBP (P = 0.860), and ΔPP (P = 0.425). When it came to four hours after the commencement of CST, we found statistical differences in ΔPP (P = 0.042) while no statistical differences were found in ΔSBP (P = 0.888) and ΔDBP (P = 0.069). It showed that the ΔPP of the 20° group (1.46 ± 10.05 mmHg) was significantly higher than that of the 40° group (-0.05 ± 11.00 mmHg).

**Table 4:** Comparison of blood pressure changes at 2,3 and 4 h after clonidine stimulation test in children with 20° and 40°

	20°	40°	*t*	*P*
*2 h*				
Δ systolic blood pressure (mmHg)	-11.99 ± 11.48	-11.33 ± 11.80	-0.815	0.415
Δ diastolic blood pressure (mmHg)	-13.08 ± 10.83	-13.37 ± 10.48	0.397	0.692
Δ pulse pressure (mmHg)	1.08 ± 10.29	2.04 ± 11.33	-1.258	0.209
*3 h*				
Δ systolic blood pressure (mmHg)	-10.57 ± 12.48	-10.09 ± 12.24	-0.555	0.579
Δ diastolic blood pressure (mmHg)	-12.56 ± 11.35	-12.70 ± 10.80	0.176	0.860
Δ pulse pressure (mmHg)	2 ± 10.56	2.61 ± 11.34	-0.798	0.425
*4 h*				
Δ systolic blood pressure (mmHg)	-7.63 ± 12.18	-7.75 ± 12.32	0.141	0.888
Δ diastolic blood pressure (mmHg)	-9.09 ± 11.66	-7.70 ± 9.80	-1.823	0.069
Δ pulse pressure (mmHg)	1.46 ± 10.05	-0.05 ± 11.00	2.039	0.042

## Discussion

In this retrospective cross-sectional study, we assessed the change in blood pressure at 1, 2, 3, and 4 h after the commencement of the stimulation tests separately. The result showed that PLR, which meant raising children’s legs above cardiac level can raise the change of their pulse pressure while keeping them in a supine position did not reach this outcome. Compared with elevated children’s legs at an angle of 40°, those who elevated their legs at an angle of 20° can better raise the change of their pulse pressure.

Several research evidence indicate that the decrease in PP may be a risk factor for the death caused by shocks with hypotension. One research on trauma patients indicated that a PP of less than 45 appears to be positively correlated with death in patients with hemorrhagic shock [11]. Another study showed a positive association between 30-day mortality and initial PP < 40mmHg in septic shock patients [12]. Low PP seemed to be dangerous for patients in shock with hypotension. This may suggest that raising the change in their pulse pressure might be a good choice to alleviate the possible consequences of the hypotension caused by CST in clinical practice.

The increase in the change of pulse pressure indicates that PLR can prompt the increase of stroke volume, which can be a great help for children with hypotension caused by clonidine. The effect of PLR has already been seen as autotransfusion [13]. Morgan et al. estimated that raising a single leg at an angle of 30° may transfuse approximately 150 mL of blood to the central circulation [14], which may increase the preload of the heart. So, the increase in stroke volume may be explained by the Frank-Starling relationship in normovolemic coronary artery disease patients which meant that the increase of the preload would strengthen cardiac contractility. This effect did not raise diverse attention to relieving hypotension, which may be attributed to its inefficiency in hypovolemic hypotension [9]. However, in normovolemic patients, raising preload can help deal with hypotension [15].

Compared with PLR, another posture called the Trendelenburg position was more widely used in relieving hypotension. PLR and Trendelenburg’s position shared the same theory in increasing stroke volume. They can both increase preload and therefore increase cardiac outcome. Bart et al. reported that PLR seemed to have long-term effects while Trendelenburg’s position only lasted for 1 min. They explained this result by lower baroreceptors and more blood accumulating in the veins, atria, and pulmonary circulation [9]. This gave us theoretical support for choosing PLR as the better posture to deal with the hypotension caused by CST. Another reason for recommending PLR as the better choice was that the Trendelenburg position would be more uncomfortable, which made it quite a challenge for children to keep this posture for 4 h.

However, we are still unable to clearly explain the difference in outcomes between the 20° and 40° groups. The 20° group had a higher change in pulse pressure 4 h after CST began compared with the 40° group. We speculated that this might involve baroreceptors. Raising legs higher, from 20° to 40°, may incur extra gravitational force and hydrostatic pressure on baroreceptors, which may cause a decrease in cardiac activity to offset the effect of autotransfusion caused by PLR [16, 17]. In addition, raising children’s legs at an angle of 40° may cause discomfort, which makes it difficult for children to keep this gesture cooperatively. Since we did not monitor children closely, they may change their posture due to discomfort.

Previous studies proposed PLR exert a transient effect on blood pressure and cardiac outcome which commonly lasted less than 45 min [13, 18, 19]. PLR has always been seen as a short-term method used for first aid or as a test for predicting fluid responsiveness. This may explain why few researchers have paid attention to its long-term effects on pulse pressure [20–22]. Our research results indicate the potential of PLR in long-term treatment for strengthening cardiac contractility to alleviate clonidine-induced hypotension. This was consistent with some research, but the quantity of these studies is low and the population sizes involved are small [9, 10]. Our study may be a good supplement for researchers who study the effect of postural characteristics on blood pressure.

Our research has several strengths. Firstly, to the best of our knowledge, this is the first study to use body position in alleviating the hypotension caused by CST. As a treatment, it is easy to implement with few side effects and little cost, which means that it can be easily popularized. Secondly, besides only paying attention to demonstrating the effects of PLR, we further explored the best angle raised for this treatment, which makes this method more practical. Thirdly, we tried to study the long-term effect of this body position which was mostly seen to be only with transient ones.

There are still limitations and deficiencies that need to be improved. Firstly, the participants only stayed in the hospital for about 3 days, which meant that they were not so familiar with the environment. This indicated that the white coat effect might not be excluded. Secondly, when the children were kept lying on their beds, we did not record whether the participants fell asleep and their sleeping time during CST was not recorded either. As sleeping may affect the patient’s blood pressure, these effects cannot be excluded. Thirdly, our study was only a single-center study which meant that this can only reflect the effects on children around Nanjing. This meant that our results may not be generalizable enough. Thus, more evidence is needed to determine whether PLR can alleviate the adverse effects of clonidine-induced hypotension.

## Conclusions

Our result indicates that elevating the foot of the beds of the patients who are undergoing clonidine stimulation tests at an angle of 20° seemed to be a good choice to alleviate the hypotension caused by the tests. Our research may give theoretical support for those who try to utilize passive leg raising or other postural characteristics to deal with the adverse effects of this test.

## Data Availability

The datasets generated during and/or analyzed during the current study are available from the corresponding author on reasonable request.

## Abbreviations

SD: Standard deviations
CST: Clonidine stimulation test
PLR: Passive leg raising
SBP: Systolic blood pressure
DBP: Diastolic blood pressure
PP: Pulse pressure
